# Vancomycin-intermediate Staphylococcus aureus in a home health-care patient.

**DOI:** 10.3201/eid0706.010618

**Published:** 2001

**Authors:** J C Hageman, D A Pegues, C Jepson, R L Bell, M Guinan, K W Ward, M D Cohen, J A Hindler, F C Tenover, S K McAllister, M E Kellum, S K Fridkin

**Affiliations:** Division of Healthcare Quality Promotion, Centers for Disease Control and Prevention, Atlanta, Georgia 30333, USA. jhageman@cdc.gov

## Abstract

In June 2000, vancomycin-intermediate Staphylococcus aureus (VISA) was isolated from a 27-year-old home health-care patient following a complicated cholecystectomy. Two VISA strains were identified with identical MICs to all antimicrobials tested except oxacillin and with closely related pulsed-field gel electrophoresis types. The patient was treated successfully with antimicrobial therapy, biliary drainage, and reconstruction. Standard precautions in the home health setting appear successful in preventing transmission.


### Vancomycin-Intermediate *Staphylococcus aureus* in a Home Health-Care Patient

**Jeffrey C. Hageman,* David A. Pegues,† Carrie Jepson,‡ Rose Lee Bell,‡ Mary Guinan,§ Kevin W. Ward,† Martin D. Cohen,† Janet A. Hindler,† Fred C. Tenover,* Sigrid K. McAllister,* Molly E. Kellum,* and Scott K. Fridkin***

*Centers for Disease Control and Prevention, Atlanta, Georgia, USA; †University of California Los Angeles Medical Center, Los Angeles, California, USA; ‡Clark County Health District, Las Vegas, Nevada, USA; and §Nevada State Health Division, Carson City, Nevada, USA

In June 2000, vancomycin-intermediate *Staphylococcus aureus* (VISA) was isolated from a 27-year-old home health-care patient following a complicated cholecystectomy. Two VISA strains were identified with identical MICs to all antimicrobials tested except oxacillin and with closely related pulsed-field gel electrophoresis types. The patient was treated successfully with antimicrobial therapy, biliary drainage, and reconstruction. Standard precautions in the home health setting appear successful in preventing transmission.

To date, four of the eight cases of infection by *Staphylococcus aureus* with reduced susceptibility to vancomycin (vancomycin-intermediate *S. aureus* [VISA] or glycopeptide-intermediate *S. aureus* [GISA]) have been reported in the United States (1-3). We report a fifth case of VISA infection in the United States, and the first to occur during home health-care therapy. While all previous VISA strains have been oxacillin resistant, one of the two VISA strains identified in this investigation was oxacillin susceptible.

### Case Report

The patient, a 27-year-old woman, was well until acute cholecystitis developed in February 2000. She had undergone left nephrectomy and radiation therapy at 4 years of age for Wilms tumor. There was no evidence of tumor recurrence, and she had normal renal function. She worked as a nurse in several long-term care facilities for 6 years.

She underwent emergency laparoscopic cholecystectomy that was complicated by laceration of the common bile duct. She required open cholecystectomy and placement of a biliary drainage tube (i.e., T-tube). Cefotetan (1,000 mg) had been given parenterally as perioperative prophylaxis. A T-tube cholangiogram performed 2 weeks after the procedure showed no common bile duct obstruction or stricture, and the T-tube was removed. During the next 2 weeks, fever and right upper quadrant abdominal pain developed. A computed tomography (CT) scan of the abdomen demonstrated multiple abscesses in right and left hepatic lobes. Endoscopic retrograde cholangiopancreatography (ERCP) demonstrated mild narrowing of the common bile duct. A biliary stent was placed and levofloxacin and metronidazole were administered, but the patient's condition did not improve. Aspiration of one abscess under CT guidance revealed no pathogens. A bile culture obtained during ERCP stent replacement identified *Candida albicans* and oxacillin-resistant *S. aureus* (ORSA, vancomycin MIC=2 µg/mL). Intravenous vancomycin, metronidazole, levofloxacin, and fluconazole were administered, and the patient was transferred from the hospital in Nevada to Los Angeles, where a transhepatic biliary drainage catheter was placed. In April 2000, she was discharged to home, where she was followed weekly by home health-care nurses.

Over the next 10 weeks, the patient received ciprofloxacin (500 mg every 12 hours), metronidazole (250 mg every 8 hours), and vancomycin (from 750 mg to 1,100 mg every day). Serum vancomycin trough levels obtained approximately once each week were from 2.7 µg/mL to 4.9 µg/mL. During elective exchange of the transhepatic biliary drain on June 6, 2000, a culture of bile drainage identified *C. albicans*, *Stenotrophomonas maltophilia*, ORSA, and VISA.

After VISA was confirmed, vancomycin therapy was stopped, and treatment was modified to include linezolid (600 mg twice a day), trimethoprim-sulfamethoxazole (160 mg and 800 mg twice a day), and doxycycline (100 mg orally twice a day). After 10 days of therapy, the patient's condition improved, and repeat culture of the bile showed no growth of *S. aureus*. Six weeks after therapy for VISA was initiated, the patient underwent reconstruction of the bile duct with a Roux-en-Y hepaticojejunostomy and removal of intrahepatic drain. During this hospitalization, therapy for VISA was discontinued. Although the VISA infection cleared, the patient continues to have symptoms of gastroesophageal reflux disease.

After 24 hours' incubation, primary isolation purity plates of biliary drainage grew large, yellow, beta-hemolytic colonies of *S. aureus* with an oxacillin MIC >16 µg/mL and a vancomycin MIC of 2 µg/mL by broth microdilution. After 48 hours of incubation, additional small colonies were visible, and two morphologically distinct strains of *S. aureus* were identified on subculture. In the clinical laboratory, vancomycin susceptibility of both strains of *S. aureus* was determined by broth microdilution (vancomycin MIC = 8 µg/mL) and was confirmed by E-test (vancomycin MIC = 6 µg/mL) and

Address for correspondence: Jeff Hageman, Centers for Disease Control and Prevention, Division of Healthcare Quality Promotion, Mailstop A35, 1600 Clifton Road, Atlanta, GA 30333, USA; fax: 404-639-2647; e-mail: jhageman@cdc.gov growth on a brain heart infusion screening plate containing 6 µg of vancomycin. These tests were repeated and confirmed on both strains at the Centers for Disease Control and Prevention.

The antimicrobial susceptibility profiles of the two strains were identical for 10 of the 11 antimicrobials tested: susceptible to gentamicin, tetracycline, quinupristin-dalfopristin, and trimethoprim-sulfamethoxazole; intermediate to vancomycin and chloramphenicol; and resistant to erythromycin, clindamycin, levofloxacin, ciprofloxacin, and rifampin. However, strain no. 1 was resistant to oxacillin (MIC >16 µg/mL) and was *mecA* positive by polymerase chain reaction (4); strain no. 2 was susceptible to oxacillin (MIC = 0.5 µg/mL) and was *mecA* negative.

Before the identification of VISA, the home health-care agency nurses caring for the patient had routinely used standard precautions (i.e., washing hands before and after contact with the patient, using gloves for contact with nonintact skin or mucous membranes, and wearing mask and eye protection if splashing was anticipated). Because the patient administered vancomycin and emptied the biliary drainage reservoir herself, nurses did not wear face shields or masks before VISA was identified. The nurses' activities generally were limited to inspecting and flushing the percutaneously inserted central venous catheter. After identification of VISA, additional measures were instituted, including care by a single designated nurse and use of gloves, mask, and gowns for all patient contact (5).

Before these precautions were instituted, we assessed possible person-to-person transmission of VISA. Cultures were obtained on dry cotton swabs from both anterior nares of the patient, of her household contacts (parents and spouse), and of the three nurses who had provided care in the previous 3 months. Each swab was plated on mannitol salt agar and incubated for 48 hours at 35°C. *S. aureus* isolates were initially screened by Staphaurex (Murex Diagnostics Inc., Norcross, GA) and identified by using standard biochemicals (6). Susceptibility testing was performed by broth microdilution (7). The patient and one of the three nurses were nasal carriers of ORSA (vancomycin MIC = 2 µg/mL). Ten days later, the nares of the carrier nurse were recultured, but no *S. aureus* carriage was detected. This health-care worker had not received topical or systemic antimicrobial therapy for *S. aureus* decolonization.

*S. aureus* strains from the patient and the health-care worker were compared by pulsed-field gel electrophoresis (PFGE) using *Sma*I- and *Eag*I-digested chromosomal DNA (Figure 1). The patient's oxacillin-resistant VISA strain was closely related by SmaI and EagI to the patient's oxacillin-susceptible strain of VISA. Although by SmaI the ORSA strain isolated from the nares of the health-care worker appeared to be related to the patient's oxacillin-resistant strain, by EagI the fragment patterns were different, and the isolates were classified as unrelated (8). Finally, the patient's oxacillin-resistant VISA strain had a similar fragment pattern by SmaI compared with the four previously reported U.S. VISA strains (2,3,9) (Figure 2).

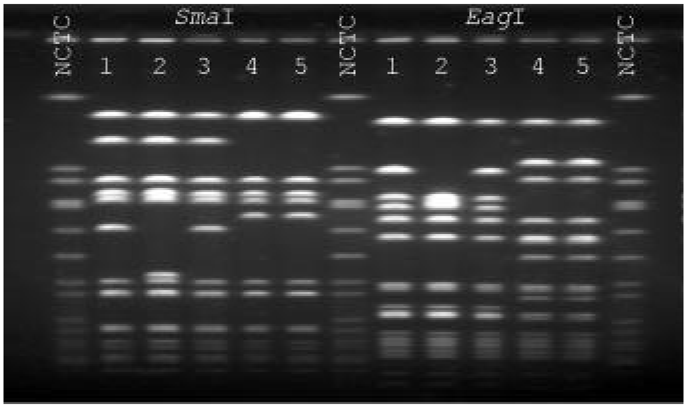

Figure 1. Pulsed-field gel electrophoresis profiles of *Sma*I- and *Eag*I-Digested DNA. NCTC, National Collection of Type Cultures 8325 control. Lane 1, patient's oxacillin-resistant vancomycin-intermediate *Staphylococcus aureus* (VISA); lane 2, patient's oxacillin-susceptible VISA; lane 3, patient's oxacillin-resistant *S. aureus* (ORSA, vancomycin MIC = 2 µg/mL) from anterior nares; lanes 4 and 5, isolates of ORSA (vancomycin MIC = 2 µg/mL) from the health-care worker's anterior nares.

### Conclusions

This case report has several unique aspects. First, each of the four previously reported U.S. patients with VISA had end-stage renal disease; this patient had normal renal function. Second, one of the patient's VISA strains did not contain the *mecA* gene and was oxacillin susceptible; previously published cases all contained the *mecA* gene. This patient's VISA strains likely emerged from the same parent strain, but one underwent a deletion to become *mecA* negative. The reasons for this are unclear and deserve further study. Third, this infection developed in the home health-care setting, not in a dialysis or acute-care setting, as did previous cases. Potential risk factors for VISA, such as prolonged exposure to vancomycin, are not limited to dialysis or acute-care settings, and clinical microbiology laboratorians and clinicians must be vigilant to recognize these strains in at-risk patients. This includes retesting *S. aureus* isolates from patients who do not respond to traditional therapy (5). Finally, this patient was successfully treated by surgery and pharmacotherapy. It is unclear if this infection would have

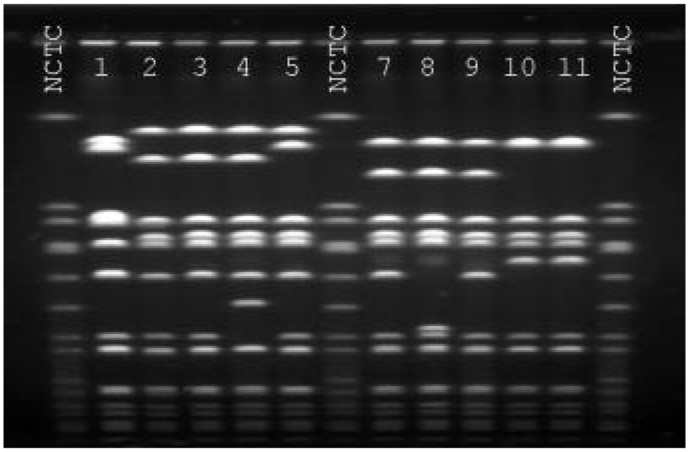

Figure 2. Pulsed-field gel electrophoresis profiles of *Sma*I digested DNA. NCTC, National Collection of Type Cultures 8325 control. Lane 1, vancomycin-intermediate *Staphylococcus aureus* (VISA) isolate from Japan, otherwise designated Mu50; lane 2, VISA isolate from Michigan (7); lane 3, VISA isolate from New Jersey (7); Lane 4, VISA isolate from New York (1); lane 5, VISA isolate from Illinois (3); lane 7, patient's oxacillin-resistant VISA; lane 8, patient's oxacillin-susceptible VISA; lane 9 patient's oxacillin-resistant *S. aureus* (ORSA, vancomycin MIC = 2 µg/mL) from anterior nares; lanes 10 and 11, isolates of ORSA (vancomycin MIC=2 µg/mL) from the health-care worker's anterior nares.

resolved with either treatment alone. The treatment of VISA infections reported to date has varied (10). The need for combination therapy is unclear, but previous reports suggest vancomycin monotherapy is inadequate (9). Until studies demonstrate efficacy of a particular regimen, clinicians must rely on the susceptibility profiles of isolates to determine which antimicrobial therapy is appropriate. Removal of prosthetic material or surgical intervention also may play an integral part in successful treatment.

Similar to previous reports, inadequate dosing of vancomycin for treatment of serious ORSA infection likely contributed to the emergence of this VISA. The limited concentration of vancomycin in bile (30% to 50% of serum levels), poor penetration of this drug into abscesses, and subtherapeutic vancomycin dosing may have favored emergence of VISA. In addition, this patient's VISA appears to have emerged from a preexisting *S. aureus* infection; however, the isolate that caused this preexisting infection was not available for study. The original ORSA isolate associated with this patient's hepatic abscess may have been acquired during her initial hospitalization or previous employment. Regardless, this VISA appears to be closely related by PFGE to the other U.S. VISA strains, suggesting certain *S. aureus* strains may have a predisposition to express the VISA phenotype.

As with previous U.S. reports, no evidence suggests that VISA spread to health-care personnel or contacts. The use of standard precautions appears to have been sufficient to prevent transmission of this strain from patient to health-care worker. All health-care agencies should use appropriate infection control practices to prevent spread of epidemiologically important organisms from patient to health-care worker and patient to patient. Having such policies in place, regardless of the setting, will protect patients and workers from unnecessary risks.

### Acknowledgments

We thank Nancy Archambeault, George Killgore, Bertha Hill, Jasmine Mohammed, Stacey Holt, and David Bruckner for technical assistance, and Pam Gopsill, Ajumobi C. Agu, Jerome Hruska, and Eugene Speck for their assistance and support in this investigation.

Mr. Hageman is an epidemiologist in the Division of Healthcare Quality Promotion, Centers for Disease Control and Prevention. His research focuses on surveillance of resistant staphylococcal infections and enhancement of national laboratory capacity to detect emerging antimicrobial-resistant organisms.

### References

1. Rotun SS, McMath V, Schoonmaker DJ, Maupin PS, Tenover FC, Hill BC, et al. *Staphylococcus aureus* with reduced susceptibility to vancomycin isolated from a patient with fatal bacteremia. Emerg Infect Dis 1999;5:147-9.
2. Centers for Disease Control and Prevention. Reduced susceptibility of *Staphylococcus aureus* to vancomycin—Japan, 1996. MMWR Morb Mortal Wkly Rep 1997;46:624-6.
3. Centers for Disease Control and Prevention. *Staphylococcus aureus* with reduced susceptibility to vancomycin—Illinois, 1999. MMWR Morb Mortal Wkly Rep 2000;48:1165-7.
4. Vannuffel P, Gigi J, Ezzedine H, Vanderman B, Delmee M, Wauters G, et al. Specific detection of methicillin-resistant *Staphylococcus aureus* species by multiplex PCR. J Clin Microbiol 1995;33:2864-7.
5. Centers for Disease Control and Prevention. Interim guidelines for prevention and control of staphylococcal infection associated with reduced susceptibility to vancomycin. MMWR Morb Mortal Wkly Rep 1997;46:626-35.
6. Kloos WE, Bannerman TL. Staphylococcus and micrococcus. In: Murray PR, editor. Manual of clinical microbiology. 7th ed. Washington: American Society for Microbiology Press; 1999. p. 264-82.
7. National Committee for Clinical Laboratory Standards. Methods for dilution antimicrobial susceptibility tests for bacteria that grow aerobically. 5th ed. Approved standard M7-A5. Wayne (PA): The Committee; 2001.
8. Tenover FC, Arbeit RD, Goering RV, Mickelsen PA, Murray BE, Persing DH, et al. Interpreting chromosomal DNA restriction patterns produced by pulsed-field gel electrophoresis: criteria for bacterial strain typing. J Clin Microbiol 1995;33:2233-9.
9. Smith TL, Pearson ML, Wilcox KR, Cruz C, Lancaster MV, Robinson-Dunn B, et al. Emergence of vancomycin resistance in *Staphylococcus aureus*. N Engl J Med 1999;340:493-501.
10. Fridkin SK. Vancomycin-intermediate and -resistant *Staphylococcus aureus*: what the infectious disease specialist needs to know. Clin Infect Dis 2001;32:108-15.
